# Vaccination Persuasion Online: A Qualitative Study of Two Provaccine and Two Vaccine-Skeptical Websites

**DOI:** 10.2196/jmir.4153

**Published:** 2015-05-29

**Authors:** Lenny Grant, Bernice L Hausman, Margaret Cashion, Nicholas Lucchesi, Kelsey Patel, Jonathan Roberts

**Affiliations:** ^1^Vaccination Research GroupDepartment of EnglishVirginia TechBlacksburg, VAUnited States

**Keywords:** vaccination, communication, Internet, social networking, Web 2.0, qualitative research

## Abstract

**Background:**

Current concerns about vaccination resistance often cite the Internet as a source of vaccine controversy. Most academic studies of vaccine resistance online use quantitative methods to describe misinformation on vaccine-skeptical websites. Findings from these studies are useful for categorizing the generic features of these websites, but they do not provide insights into why these websites successfully persuade their viewers. To date, there have been few attempts to understand, qualitatively, the persuasive features of provaccine or vaccine-skeptical websites.

**Objective:**

The purpose of this research was to examine the persuasive features of provaccine and vaccine-skeptical websites. The qualitative analysis was conducted to generate hypotheses concerning what features of these websites are persuasive to people seeking information about vaccination and vaccine-related practices.

**Methods:**

This study employed a fully qualitative case study methodology that used the anthropological method of thick description to detail and carefully review the rhetorical features of 1 provaccine government website, 1 provaccine hospital website, 1 vaccine-skeptical information website focused on general vaccine safety, and 1 vaccine-skeptical website focused on a specific vaccine. The data gathered were organized into 5 domains: website ownership, visual and textual content, user experience, hyperlinking, and social interactivity.

**Results:**

The study found that the 2 provaccine websites analyzed functioned as encyclopedias of vaccine information. Both of the websites had relatively small digital ecologies because they only linked to government websites or websites that endorsed vaccination and evidence-based medicine. Neither of these websites offered visitors interactive features or made extensive use of the affordances of Web 2.0. The study also found that the 2 vaccine-skeptical websites had larger digital ecologies because they linked to a variety of vaccine-related websites, including government websites. They leveraged the affordances of Web 2.0 with their interactive features and digital media.

**Conclusions:**

By employing a rhetorical framework, this study found that the provaccine websites analyzed concentrate on the accurate transmission of evidence-based scientific research about vaccines and government-endorsed vaccination-related practices, whereas the vaccine-skeptical websites focus on creating communities of people affected by vaccines and vaccine-related practices. From this personal framework, these websites then challenge the information presented in scientific literature and government documents. At the same time, the vaccine-skeptical websites in this study are repositories of vaccine information and vaccination-related resources. Future studies on vaccination and the Internet should take into consideration the rhetorical features of provaccine and vaccine-skeptical websites and further investigate the influence of Web 2.0 community-building features on people seeking information about vaccine-related practices.

## Introduction

### Background

Current concerns about vaccination resistance often cite the Internet as a source of vaccine controversy. Despite the United States’ high vaccination rates among children and adults, physicians and researchers have perpetuated the belief that vaccine-skeptical websites contribute to lower levels of vaccination among children by effectively persuading parents against immunizing their children. Websites promoting vaccine-skeptical discourses are scrutinized routinely in the academic literature; however, the preponderance of this research aims at demonstrating that the information they circulate is inaccurate and deceptive to visitors seeking information on vaccines and vaccination-related practices. It is true that as a result of these studies, the medical community has gained a greater understanding of the types of information presented on vaccine-skeptical websites and deeper insights into how these websites deploy this information to make persuasive arguments against vaccines and vaccination. The majority of academic studies of vaccine-skeptical websites use quantitative methods to taxonomize arguments against vaccination on these websites. Although this information is useful for categorizing their generic features, it has not provided insights into why these websites successfully persuade their viewers.

To date, there has been no attempt to understand the qualitative features of vaccine-skeptical websites. The research presented in this paper attempts to fill this gap by employing a case study approach to a smaller number of websites than is typical of quantitative studies of vaccine skepticism on the Internet. In addition, this study examines both vaccine-skeptical and vaccine-promoting websites to compare the rhetorical features through which they attempt to reach their audiences. By deploying a qualitative methodology, researchers can better understand the rhetorical features of both types of websites. As a result of this study, we can better understand the specific mechanisms by which vaccine-skeptical organizations have been able to use the Internet to successfully spread their messages.

### Literature Review

Since the United Kingdom passed the Vaccination Act of 1853, vaccine-skeptical groups have leveraged the available means of persuasion to voice their opposition to compulsory vaccination. Some groups resorted to public demonstrations, legal actions, and the occasional riot after the passage of the 1853 law, but others, such as the Anti-Compulsory Vaccination League, which formed in response to the 1867 Vaccination Act, found publishing their ideas in newsletters and journals to be a more effective means of responding to government vaccine mandates for children [1]. Other groups followed suit. The *Anti-Vaccinator* journal was founded in 1869 followed by the *National Anti-Compulsory Vaccination Reporter* in 1874 and the *Vaccination Inquirer* in 1879 [2]. After a smallpox epidemic in the 1870s, US vaccine-skeptical movements circulated pamphlets and journals in response to state attempts to pass new vaccine legislation or enforce extant laws. During the Progressive Era, regional antivaccination movements, such as the one in Portland, Oregon, assumed the political mantle of the populist democracy movement [3]. At that time, resistance to vaccination in the United States took 2 dominant forms: ordinary Americans who resisted compulsory vaccination and self-identified antivaccination activists who joined societies, wrote newsletters, and were largely middle class [4]. Political opposition to vaccine mandates and the circulation of populist information continued throughout the 20th century, although it changed as a result of the rapid development of many vaccines in the second half of the century.

In the late 20th century, the Internet transformed mass communication, affording its users new means of sharing information, forging interpersonal connections, and establishing association [5-8]. The relatively short history of the Internet can be divided into 2 epochs: Web 1.0, which emerged in the 1980s, and Web 2.0, which emerged in the mid-2000s. Web 1.0 is characterized by static webpages that display information [9,10] and text-based online virtual communities where users interact with one another on topics of mutual interest [6,8,11]. Web 1.0 also introduced hyperlinking, the now-familiar clicking process that redirects a Web user to another website. One static website could be hyperlinked to another for a myriad of rhetorical purposes, including demonstrating affinity, offering additional information, or leveraging another website’s credibility [7]. Web 2.0 is best characterized as a platform for Internet applications that afford users the ability to “harness collective intelligence” [9]. Web 2.0 permits users to generate and post their own content and comment on what others have shared [9,10]. One of its most defining characteristics is that it affords 2-way communication via social media, such as blogs, Facebook, Twitter, YouTube, and other websites. Where the static Web pages of Web 1.0 allowed unidirectional communication (a user reading text on a screen), Web 2.0 promotes interactivity between users who can easily respond to one another via text and images.

The Internet and Web 2.0 have changed the way that people access health information. Ordinary people have greater access to medical information [12] and online patient communities have organized on websites [13] and social media [14] to provide information and support for many diagnoses. Easy access to information has led to both self-diagnosis and self-doctoring [15]. According to Pew Research’s Health Online 2013 poll, 72% of Internet users surveyed looked for health information online and 35% opted to self-diagnose with Web-based information rather than visit a clinician [16]. It is estimated that 16% of those seeking online medical information searched for vaccination information, with 70% of this group stating that their findings influenced their vaccine decisions [17]. In addition to peer-reviewed medical information, Internet users also have access to health information generated by nonmedical practitioners, which has raised concerns about the quality of online medical information available on the Internet [18].

### Online Vaccination Skepticism and Web 1.0

Current accounts of vaccine skepticism tend to identify its origins in the present period with the circulation of information on the Internet (eg, Kodish [19]). Although early proponents of the Internet saw its potential as a means of promoting democracy through the circulation of information [8,20], others viewed the Internet as a “Pandora’s box” of misinformation [21]. As Internet use proliferated at the end of the 20th and beginning of the 21st centuries, researchers began to pay attention to the World Wide Web as a site of information dissemination for vaccine skeptics. Many of these studies employed a Web 1.0 understanding of online communication even after Web 2.0′s social media paradigm was well in place (ie, they conceived the Internet as a repository of information and not a dynamic space were users interact with one another). The main objectives of these studies were to ascertain the philosophies of so-called antivaccination websites and point out the misleading or inaccurate information they circulated in cyberspace. A number of these studies created taxonomies or tried to identify specific features of the misunderstandings that these sites were thought to perpetuate. The article that best exemplifies this tendency is Jacobson et al [22], the title of which is indicative of the approach: “A Taxonomy of Reasoning Flaws in the Anti-Vaccine Movement.”

During this period, 2 studies about vaccination influenced by the Pandora’s box metaphor appeared in the pages of medical journals [23,24]. Taking as his exigence the concern that vaccine-skeptical groups were using the Internet to gain political momentum in the United States and Western Europe, Nasir [23] analyzed 51 websites that opposed routine childhood vaccination, addressing content, common themes, philosophy, links to other websites, and strategies to avoid routine immunization. Although the websites promoted a variety of philosophies, they exhibited some commonalities: they listed adverse effects of vaccines and presented themselves as unbiased toward vaccination [23]. Nasir found that clicking deeper into the websites revealed a strong bias against vaccines and vaccination and concluded that the availability of vaccine-skeptical information on the Internet is troublesome. Nasir expressed concern that Web surfers are ill equipped to assess its reliability, an argument that is nearly ubiquitous in subsequent studies of vaccine skepticism on the Internet [23].

Two years later, Davies et al [25] examined the content of 100 similar websites from a rhetorical perspective to better understand the social discourses in which vaccine-skeptical claims are embedded. Their rhetorical analysis revealed that vaccine-skeptical websites portrayed themselves as authorities on vaccination, appealed to viewers’ emotions through personal testimonies of vaccine injury and calls for parental responsibility, and maintained a discourse of truth seeking often advancing evidence of medical conspiracies bolstered by their own privileged information. Davies and colleagues caution medical practitioners from refuting vaccine-skeptical discourses based solely on “the facts,” suggesting instead that provaccination websites employ emotional counterappeals featuring “images and stories of children harmed by vaccine-preventable illnesses” [25].

Another study by Wolfe et al [26] made similar observations in its analyses of the content and design attributes of 22 vaccine-skeptical websites. From their content analyses, they found that all websites in their sample expressed “a variety of claims that are largely unsupported by peer-reviewed scientific literature,” including themes of concern about vaccine safety and efficacy, “governmental abuses” of civil liberties, and preferences for alternative (nonbiomedical) health practices. Their analyses of the websites’ design attributes resulted in a list of 10 common themes that, from a rhetorical perspective, conflate content (narratives of parents of vaccine-injured children), digital ecology (the content to which the website links), visual rhetoric (images of “scary needles” and “harmed children”), and commerce (solicitations of donations and merchandise for sale) [26]. The authors do note that defining what counts as content on a website is “a problem” [26]. Such a problem is likely to occur when websites are treated like pages in a book rather than interactive spaces where users connect to share experiences, expertise, and interpretations of information.

### Online Vaccine Skepticism and Web 2.0

The ascendency of social media in the mid-2000s adds another layer of complexity to online vaccine discourses. The multimedia nature of Web 2.0 websites allows vaccine-skeptical groups a means of constructing more sophisticated arguments than a single medium could afford. One study notes that antivaccine movements are well versed in multimedia communication because the groups often are led by spokespersons who use a variety of media (eg, books, television appearances) to build their ethos (credibility) as whistleblowers [27]. Although researchers have created sophisticated taxonomies of static websites [22,28,29], the strategies they offer to counter vaccine-skeptical discourses either have not been adopted by provaccine websites or have not been effective in general. For instance, one strategy offered is mass education campaigns that share images and personal narratives of people affected by vaccine-preventable diseases, such as pertussis [28]. They also suggest communicating statistics that demonstrate how vaccine-preventable diseases increase as vaccination rates decline. Using scare tactics and arguing about facts has not proven to be an effective strategy for making vaccine-skeptical parents amenable to childhood vaccination [24,30]. One main reason is the social networking features of Web 2.0 [31] that transform static Web pages into information hubs where viewers can share personal experiences in the form of images and narrative to create or participate in a community with individuals who share their vaccination beliefs.

Web 2.0′s social networking capabilities have aided health communicators in targeting messages to specific audiences [32] and helped patients and medical practitioners to gather information about diseases and diagnoses [27]. Although social networking technologies make it easy for users to crowdsource information, there is widespread concern about the quality of the information that is circulated among users and the extent to which that information influences people’s decisions to vaccinate themselves [33] and their children [34]. As users grow more accustomed to Web 2.0 technologies, it becomes more difficult to impose the authority of establishment medicine on online discourse. Witteman and Zikmund-Fisher [35] suggest “in this Web environment, effective communication about vaccinations is not about controlling what is available but rather, it is about responding and participating in an interactive, user-responsive environment.” To this end, a growing number of studies attempt to understand the flow of information on specific Web 2.0 sites.

Research on vaccination and Web 2.0 suggests that websites featuring user-generated content are more likely to support vaccination viewpoints that counter or question medical science [36]. Venkatraman et al [36] found that websites that support greater freedom of speech (ie, the website’s content is not moderated, edited, or peer-reviewed), such as YouTube and Google, are more likely to contain antivaccination content than moderated websites such as Wikipedia and PubMed. Another study analyzed nearly 40,000 opinionated Twitter users’ posts about the H1N1 vaccine and found that more information was circulated among users who shared the same positive or negative sentiments about the vaccine [37], suggesting that social media is more of an echo chamber for circulating opinions among like minds than a means of randomly influencing less opinionated users. A study of 172 YouTube videos about the human papillomavirus (HPV) vaccines found that slightly more than half of the videos expressed explicitly negative sentiments about the vaccine and that negative videos garnered a higher number of average likes than videos endorsing the HPV vaccine [38]. Compared to previous studies of HPV vaccines on YouTube, which found that approximately one-quarter [39] to approximately one-third [40] of videos opposed the HPV vaccine, Briones et al’s [38] findings suggest that vaccine critics are more effective than vaccine promoters at using social media to communicate their messages. It is also worth noting that the shift from majority positive HPV vaccine sentiments to majority negative occurred in fewer than 5 years. The relatively short time span in which attitudes change also appears to be a feature of Web 2.0, where private and public discourses about vaccines can spread virally around the Internet [31].

In an effort to counter the rhetorical efficacy of online vaccine skepticism [25], provaccine researchers have developed a 2-pronged approach that is grounded in earlier Internet studies. It begins by first attributing contemporary vaccine skepticism to Wakefield et al’s [41] now discredited claim that the measles, mumps, and rubella (MMR) vaccine contributed to the development of autism in children and then calls for the medical community to do a better job of communicating accurate medical information about childhood vaccination [42-44]. This 2-step maneuver attempts to deny the premise of vaccine skepticism through a *reductio ad absurdum* argument and creates a space for new, more accurate facts to fill the social vacuum. This tactic seems logical to vaccine proponents, but it appears to be ineffective. Although some research suggests that psychological investments may be the cause of entrenchment in antivaccine positions [45], another reason may be that vaccine-skeptical discourses predate the Wakefield debacle [46]. After all, many 21st-century arguments against vaccines are rhetorically similar to discourses in the 19th and early 20th centuries [26,28,47].

### Online Vaccination Skepticism and Postmodern Medicine

The strategy of correcting vaccine-skeptical beliefs appears to be based on a misreading of both the context of and reasons for those views. Public health attempts to correct so-called flawed reasoning are inadequate in the full context of vaccine skepticism in culture [48,49]. Hobson-West’s [30] study found that “vaccine-critical groups” tend to be differently oriented to issues of vaccination, with “radical” groups outright rejecting vaccine and “reformist” groups seeking changes to vaccination policy [30]. Both groups distrust provaccine discourses and policies and, as a result, they have reframed the notion of risk to be incommensurable with medicine’s traditional understanding [30]. Similarly, recent research suggests that corralling all discourse that does not promote vaccination under the big tent of the “antivaccination movement” collapses the variety of critical stances on vaccination [48,50-52]. Terms such as “vaccine selective” [50], “vaccine resistance” [51], and “vaccine hesitancy” [52] are used to reflect a spectrum of orientations rather than the catch-all “antivaccination.” We prefer the term “vaccine skeptical” because it denotes a variable attitude toward vaccines and vaccination versus a term, such as vaccine resistance, which forefronts an action taken against vaccines.

Beyond rejecting or reframing provaccine discourses, vaccine-skeptical websites do not subscribe to one notion of the truth; therefore, these websites’ adherents do not seem to be persuaded by claims that their beliefs are misinformed [45]. Under the current postmodern medical paradigm [53], doctors are no longer the sole arbiters of authoritative information about health and healing. The expectations that patients should inform themselves to take charge of their health decisions has resulted in “new priorities for health care” [54], such as medicine based on both social values and empirical evidence, an increased emphasis on the risks of treatment, and informed patients taking charge of their own health care decisions [53]. Kata [54] has articulated the relationship between postmodern medicine and Web 2.0 as one of flattened hierarchies where “infinite personal truths presented online are each portrayed as legitimate, thus supplanting the primacy of medical facts with a multiplicity of personal meanings and ways of knowing.” Thus, vaccine-skeptical groups appear to use the Internet to leverage postmodern notions of truth that are based on their own experiences with vaccines and their own understandings of medical science. Within the postmodern paradigm, the knowledge they generate and circulate online is not easily dismissible by attempts to better educate the public about vaccination.

## Methods

### Overview

Previous studies of vaccination information websites have taken objective approaches to locating websites via search engines. These methods included gathering and examining websites based on keyword searches. We opted for a fully qualitative case study methodology, choosing to carefully review the rhetorical features of 1 provaccine government website, 1 provaccine hospital website, 1 vaccine-skeptical information website, and 1 vaccine-skeptical website focused on a specific vaccine. The websites selected for analysis were the US Department of Health and Human Services (HHS) vaccine website Vaccines.gov [55], the Children’s Hospital of Philadelphia (CHOP) Vaccine Education Center (VEC) [56], National Vaccine Information Center (NVIC) [57], and SANE Vax, Inc [58], respectively.

### Website Selection

The websites were chosen specifically for their representativeness of specific positions in the current vaccination controversy—their choice was deliberate, not random, to demonstrate proof of concept in this pilot study. Both the Vaccines.gov and VEC websites are targeted to the general public and meant to educate. They were chosen as the representative provaccine websites because they are the US federal government’s website for the education of its citizens and a hospital-based educational site developed overseen by one of the most prominent medical proponents of vaccination, Dr Paul A Offit [59-61]. The vaccine-skeptical websites included the most established vaccine-skeptical organization (NVIC) which began in the early 1980s as Dissatisfied Parents Together [57] and a newer organization targeting concerns about the HPV vaccine, SANE Vax. Opposition to the HPV vaccines Gardasil and Cervarix has coalesced around specific injury narratives [62], and SANE Vax is one of the prominent Web venues proffering a space for these discourses. Choosing these specific websites allowed us to focus on the specific rhetorical features of each website to determine if the provaccine and vaccine-skeptical sites differed in this regard.

### Data Acquisition

To gather information from the websites, we adapted the qualitative research method of thick description to the online environment. Thick description requires the researcher to pay close attention to the contextual aspects of a research setting including minute details of the setting, the social events taking place therein, and the behaviors of the participants [63]. As a means of controlling data acquisition for consistency across the 4 websites, 5 categories of analysis were developed: information about the websites’ owners, the visual and textual content of websites, user experience, hyperlinking, and social interactivity within the website. Each of these categories corresponds to a different rhetorical element of effective communication with respect to the interactive nature of Web 2.0.

### Digital Ecologies

Aristotelian rhetoric holds that 3 modes are necessary for persuasion to take place [64]. These features are ethos, pathos, and logos. *Ethos* refers to the character of the speaker who attempts to persuade an audience. *Pathos* is the manner in which the speaker appeals to the audience’s emotions. *Logos* refers to the types of information a speaker uses to make an argument. These modes linked to the 5 categories of analysis in the following way. The website’s ownership and hyperlinks to other websites determined its ethos. The visual and textual content was the website’s logos. Social interactivity and user experience lent to the website’s pathos. Taken together, these features contributed to the website’s rhetorical efficacy.

The theoretical framework that guided this study took these Aristotelian rhetorical elements as an analytical starting place. In the second half of the 20th century, rhetoricians came to understand that persuasion is situational [65-68]. Theorists first formulated the rhetorical situation as a response to a problem, or exigence, in the world that commanded a person to communicate to change it [65]. Yet despite the robust, multifactorial nature of theories of the rhetorical situation, such a framework cannot account for the fluidity of rhetoric in networked environments. To address this shortcoming and to create a notion of rhetoric that accounted for the interconnectedness of human communication and the viral circulation of information, Edbauer [69] developed the concept of rhetorical ecologies. In a rhetorical ecology, rhetoric is not limited to a taxonomy of tropes; instead, rhetorical ecologies enable the flow information from one part of an ecosystem, such as the Internet, to another.

Because the viral circulation of information is not bounded by specific media in Edbauer’s model, we followed the lead of scholars of digital rhetoric who examined ecologies in online spaces, such as websites and gaming platforms [70,71]. Throughout this paper, we employ the term “digital ecology” to mean the discursive connections created and propagated by a website. There are 2 benefits to using the term digital ecologies to refer to rhetorical ecologies within digital spaces. The first is that the term suggests the active engagement of readers of online discourse as well as underscoring the rhetorical nature of hyperlinking [72]. The second benefit of using a term such as ecology to describe online activity is that it recalls ecosystems in nature. A website, through its links to other websites and its interactive features, can be analyzed by its size (the number it links it contains) and its diversity (whether it is open to discourses from vantage points other than its own or closed to differing opinions). For Web 2.0, an ecological model addresses the fact that the quality of information alone is insufficient to persuade someone. Rather, persuasion is effected by the information, where it is found online, how the user interacts with that information, how that information interacts with other information, and the community surrounding it.

### Usability

In considering these factors, this study also took the usability of vaccine websites into account. Usability studies are traditionally focused on making a product or application more functional for the end user [73]. When applied to online health information, a usability perspective can highlight the ways that Web design and content presentation can deny users access to information because a website is visually overwhelming, difficult to navigate, or written in such a way that it misses its target audience [74,75]. Although the prime objective of usability studies is ease of use [76], the straightforward transmission of information online has the potential to make Internet users “passive consumers of digital content” [77]. More recent studies of the usability of websites evaluated the usefulness [78,79] of websites based on the website’s ability to facilitate inquiry about the topic at hand, promote collaboration between the website’s users, and offer a multidimensional perspective that extends beyond the mere transmission of information. For the purpose of our study, we used the website usability guidelines available at Usability.gov [80] because it incorporated aspects of both ease of use and usefulness; in addition, it provided the guidelines that the federal government uses itself to evaluate website information and user experience.

### Data Analysis

Data analysis was conducted by a team of 4 advanced undergraduate researchers, who participated in Virginia Tech’s Vaccination Research Group. Each researcher was assigned a website and asked to conduct 5 rounds of observation using the thick description criteria. After each round, the group convened to discuss the findings and develop an initial analysis. Through this iterative process, each researcher synthesized his or her findings into a preliminary report with brief conclusions. These reports were a starting point for the final analysis of each case as the primary author went back to each website to confirm the findings, deepen the interpretation, develop conclusions, and write the article.

## Results

### Overview

The results are brief descriptions of the websites examined in our study. Websites are content-rich, interactive genres that do not easily lend themselves to concise textual description. Rather than offering in-depth descriptions of all aspects of each website, we present 4 case studies of the salient features of Vaccines.gov [55], VEC [56], NVIC [57], and SANE Vax [58].

### Case Study 1: Vaccines.gov

**Figure 1:**
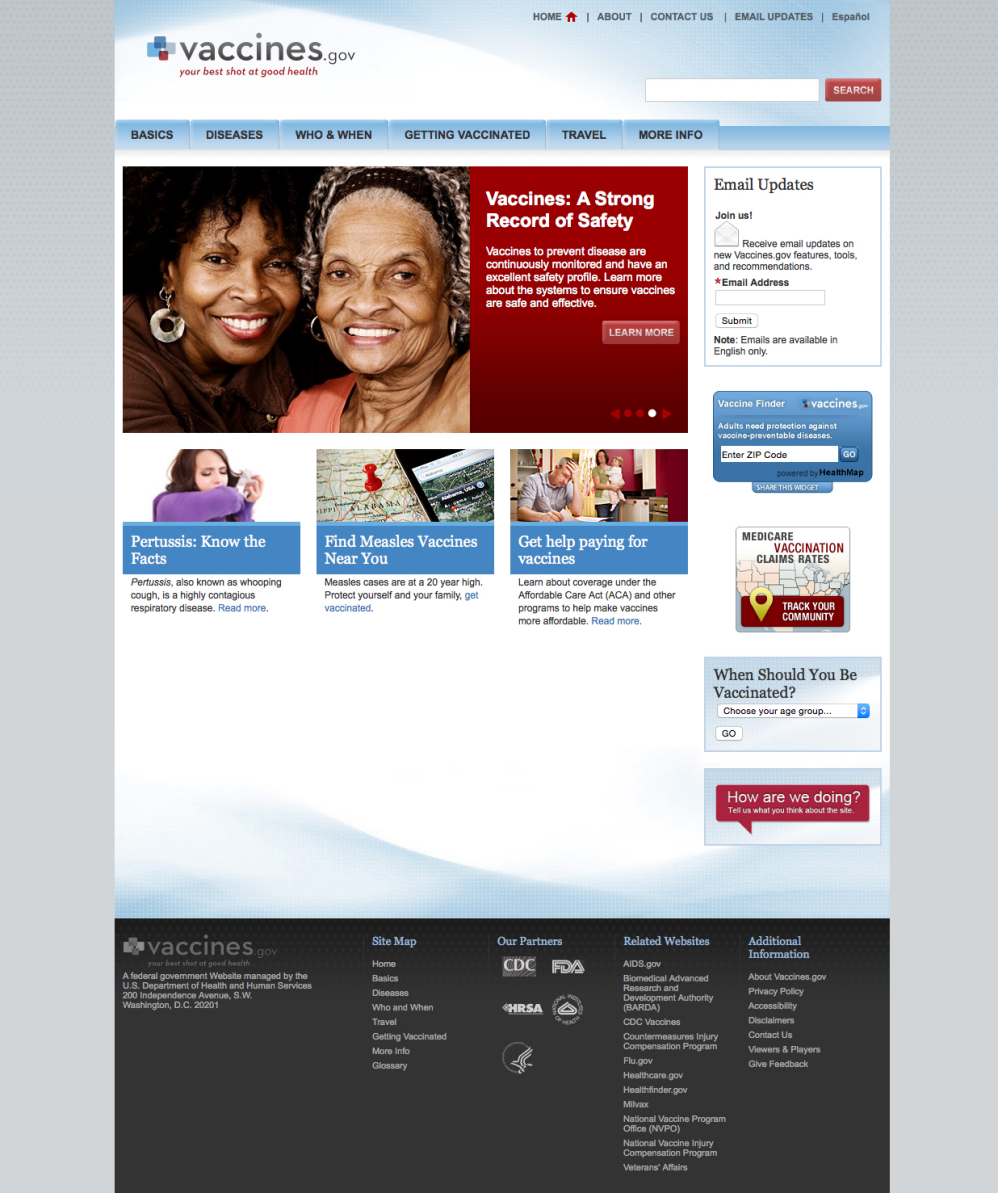
Screenshot of the Vaccine.gov home page.

The US federal government’s omnibus website, Vaccines.gov [55], bills itself as a “gateway” to information on vaccines and immunization for infants, children, teenagers, adults, and seniors” (Figure 1) The HHS National Vaccine Program Office (NVPO) coordinates the website and its content, which is created by US federal agencies including the Food and Drug Administration (FDA), Health Resources and Services Administration, National Institutes of Health, HHS, and NVPO. The intended audience of Vaccines.gov is the US general public and, as per federal mandate, the website is designed to be accessible to individuals of varying levels of literacy and ability [81]. Although all information on the website is sanctioned by the US federal government, the website carries several disclaimers, stating that the “site is not intended to be a substitute for professional medical advice, diagnosis, or treatment” and advising viewers to seek the advice of physicians and qualified health care providers regarding any questions or health concerns they may have.

Vaccines.gov contains general information about vaccines and vaccination-related practices, as well as vaccine-specific information for 22 diseases. General information is listed under 2 separate tabs on the website’s toolbar. The “Basics” tab contains information on the safety of vaccines, the efficacy of vaccines, prevention of diseases, and community (or herd) immunity. The “Getting Vaccinated” tab contains information about what children and adults can expect during vaccination, an interactive section in which visitors can enter their zip codes to find providers of adult vaccines near them, an interactive map of the United States that links to each state’s department of health, and information on how to pay for vaccinations with the Affordable Care Act and the Centers for Disease Control’s (CDC) Vaccine for Children Program. There is also a separate “Travel” tab on the navigation bar with information that links to the CDC Travel Health site.

Vaccine-specific information is categorized under 2 tabs: “Diseases” and “Who and When.” Under the “Diseases” tab, 22 vaccine-preventable diseases are listed. Each disease has its own page, most with subsections with information about the disease, information on its respective vaccine, and a tab labeled “Take Action” that includes additional government information about the disease and resources for finding where to get vaccinated. The “Who and When” tab contains vaccination schedules for 7 specific populations: infants, children, and teens aged 0 to 18 years; the Catch-up Schedule for Children aged 4 months to 18 years; college and young adults aged 19 to 24 years; adults aged 19 and older; seniors aged 65 years and older; pregnant women; and persons with health conditions.

Vaccines.gov is predominantly text based and all information references either government or scientific literature. The website also includes a limited number of images, videos, spreadsheets, and an infographic. Images feature most prominently on the website’s landing page, where they serve to illustrate the seasonal content Vaccines.gov promotes. The videos embedded on its “Features: News & Video” page offer flu vaccine information targeted at a variety of audiences, such as cartoons about the flu shot for children and scientific simulations of how the disease spreads for adults. All 19 videos are produced by government agencies.

As a repository of US federal government vaccine information, Vaccines.gov links exclusively to federal and state government websites. Although hyperlinks are numerous, the website functions as a hub for vaccine information within a relatively small network of websites.

Vaccines.gov’s limited social interactivity mirrors the website’s small digital ecology. The website’s sole interactive feature is a checkbox at the bottom of each page that asks the user “Was this page helpful?” The results of these page-by-page surveys are not available on the website, so there is no means for a user to see the feedback left by others. Additionally, the website does not include any functions that would permit users to communicate directly or indirectly with one another.

Using the guidelines published on the US federal government’s encyclopedic usability website, Usability.gov [80], as a heuristic, Vaccines.gov is best characterized as a website that employs a subject organizational scheme that organizes its content according to a variety of topics while also supporting task-oriented navigation. Visually, the website is uncluttered and easily legible due to its predominantly black text on a white background.

### Case Study 2: Children’s Hospital of Philadelphia Vaccine Education Center

**Figure 2:**
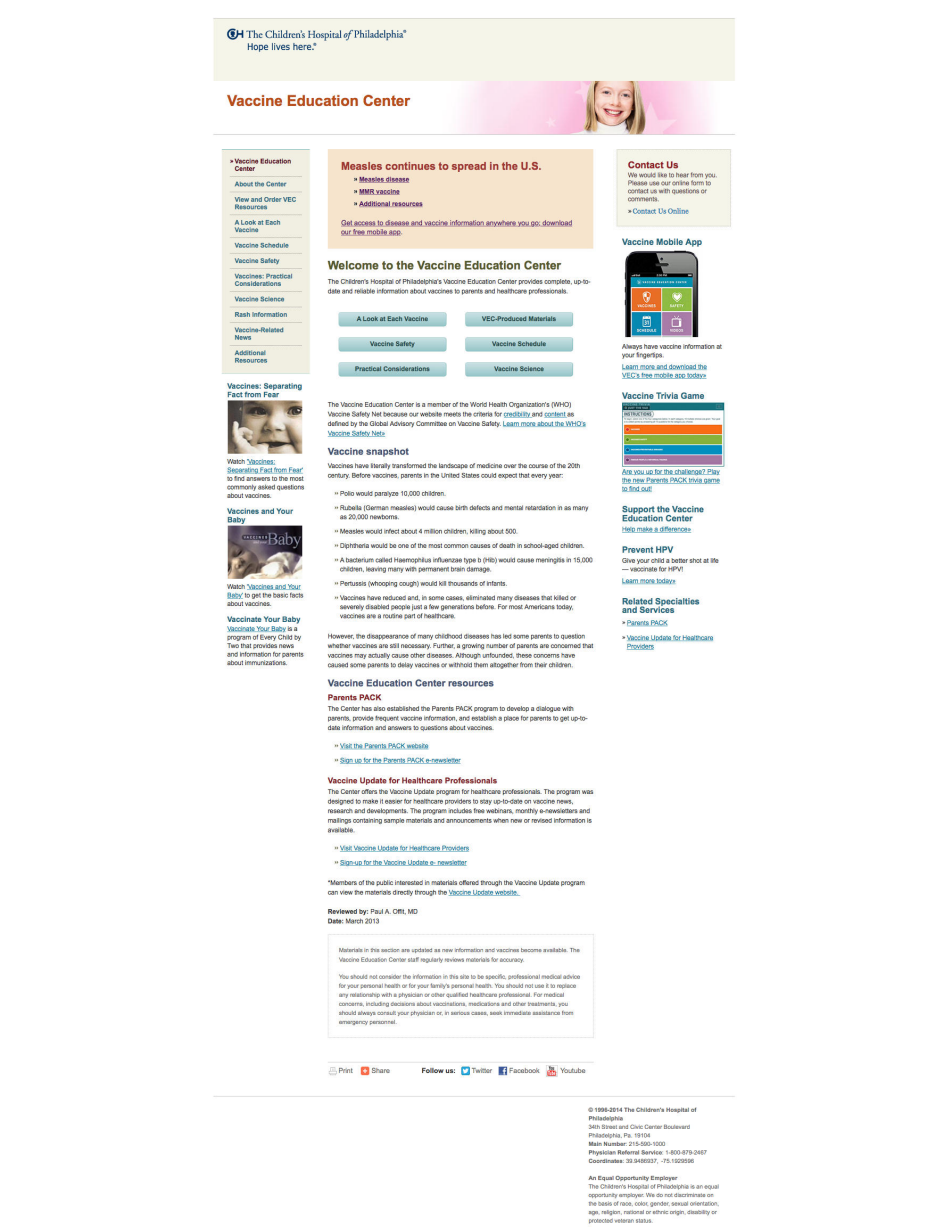
Screenshot of the Vaccine Education Center home page.

The VEC website was launched by CHOP in 2000 to “provide accurate, comprehensive, and up-to-date information about vaccines and the diseases they prevent to parents and health care professionals” [32] (Figure 2). The website’s main goal is to correct “misinformation” and “misconceptions” about vaccines and vaccination practices. The VEC is a member of the World Health Organization’s Vaccine Safety Net “because its website meets the criteria for credibility and content as defined by the Global Advisory Committee on Vaccine Safety” [56].

On the “About” page, VEC discloses that funding for the website comes entirely from CHOP and not from “vaccine manufacturers.” Visitors have the option to donate to the CHOP Foundation via a button on the main CHOP website; however, there is no donation link displayed on the VEC website itself. Additionally, the “About” page provides short biographies of the “team of scientists, physicians, mothers, and fathers” who administer, advise, and staff the VEC. The website also carries the disclaimer that none of its information is intended to be patient-specific or replace the viewer’s relationship with a qualified health care professional.

The VEC website contains information on 21 individual vaccines and 9 combination vaccines. Information on each vaccine is accessible either through the website’s sidebar or through a cluster of buttons within body of the landing page. Clicking on the “A Look at Each Vaccine” button in either location directs the viewer to a page with specific information about the vaccine and its corresponding disease. The pages are structured in a question-and-answer format, with questions moving from generic inquiries about what the disease is and how it is contracted to more population-specific questions. For instance, clicking on “Anthrax Vaccine” displays the question “Why should military personnel be vaccinated?” Similarly, “Meningococcus Vaccine” contains information targeted at college students.

The VEC uses a variety of textual and visual genres to provide information to visitors. Its landing page features links to 2 videos about infant and childhood vaccination, as well as downloadable materials for parents and health care providers. In addition to information on each vaccine, the website’s sidebar offers many other resources including vaccine schedules and vaccine safety information. There are also other scientific resources under tabs labeled “Vaccines: Practical Considerations,” “Vaccine Science,” and “Rash Information.” Information on all these pages is accompanied by references to scientific publications. The “Vaccine-Related News” tab directs users to information and resources from the CDC. Other than images of the CHOP app, links to downloadable documents, and links to videos, only one static image is used throughout the website. The VEC banner features a tightly cropped headshot of a smiling girl accented by a pink background with stars.

All content on the VEC website is created by the organization, including links to scholarly and popular press publications by the VEC’s staff, and all pages within the website are reviewed and dated by the VEC director, Dr Paul A Offit. Although the vast majority of hyperlinks direct the viewer to content within the VEC website, there are external links to “Professional and Parent Groups,” “Resources for Kids and Teens,” and “Further Reading” on the “Additional Resources” page. There are also downloadable PDF versions of CHOP’s booklets, pamphlets, and other brief communications in both English and Spanish.

The VEC website does not offer users any means of interacting with one another within its pages. Each page on the VEC website contains links to CHOP’s Twitter, Facebook, and YouTube sites; however, the content of these pages informs visitors about CHOP in general and is not specific to the VEC.

Although there is no social networking capability on the VEC website, it does offer some Web 2.0 features, such as an email newsletter, games, and a mobile app. The mobile app, called “Vaccines on the Go: What You Need to Know,” is available on both iOS and Android platforms. In addition to content from the VEC website, it includes “[a] place to save questions for the next doctor’s visit” and gives users “[t]he opportunity to easily email the VEC for answers to vaccine-related questions.”

Much like Vaccines.gov [55], the VEC website employs a combination topic and task schema. The website is easily legible with its use of black text on a white background; soft accent colors indicate items that can be clicked for more information. Its streamlined design omits a navigation bar; therefore, more content appears on screen. Despite the lack of this typical feature, its sidebar-content-sidebar layout makes the site easily navigable. The website extends its utility through its numerous downloads, which can be read offline.

### Case Study 3: National Vaccine Information Center

**Figure 3:**
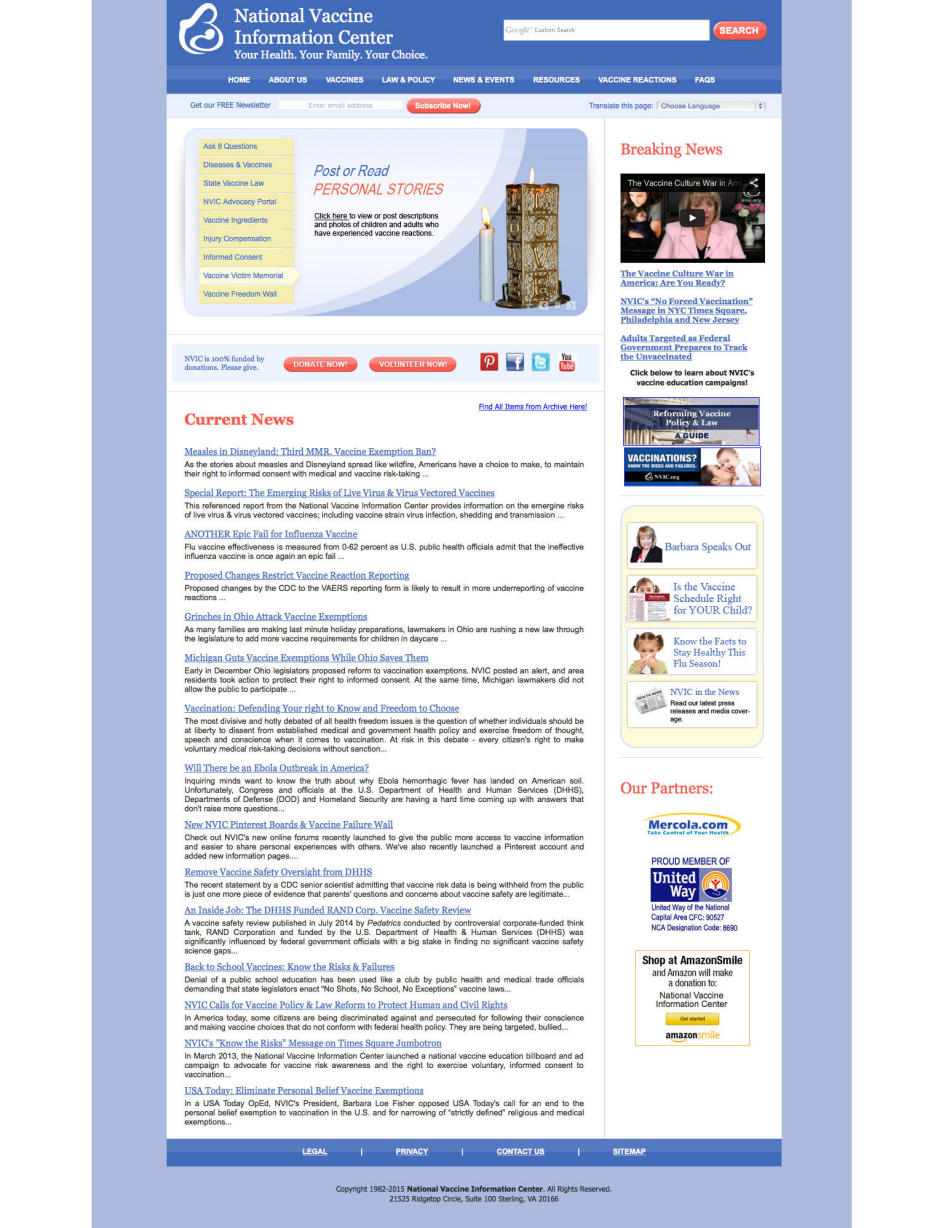
Screenshot of the National Vaccine Information Center home page.

A nonprofit organization, NVIC describes itself as “the oldest and largest consumer-led organization advocating for the institution of vaccine safety and informed consent protections in the public health system” [57] (Figure 3). NVIC states that its mission is to prevent vaccine-related injuries and deaths through public education and to promote informed consent in medicine. Additionally, NVIC funds research on vaccines and vaccination and “provides assistance to those who have suffered vaccine reactions.”

Barbara Loe Fisher, NVIC’s founder, has a significant presence on the website because the current organization grew out of her advocacy against childhood vaccines in the 1980s. Fisher’s commentary runs throughout the website and much of the site is dedicated to documenting her past and present advocacy efforts. Although her presence is ubiquitous on the website, NVIC includes graphical links to its 2 partner organizations: Mercola.com, a self-described natural health information website, and the United Way of the National Capital Area.

The landing page of the NVIC website is separated into 2 columns under a navigation bar. Atop the broad left column headed is a set of links imbedded in a rotating picture box displaying links to the website’s subsections and a right side bar with Breaking News. Current News fills up the lower left-hand side. Along the bottom of the banner at the top of the page, a series of navigation tabs lead the user into the site: “Home,” “About Us,” “Vaccines,” “Law and Policy,” “News and Events,” “Resources,” “Vaccine Reactions,” and “FAQs.” “Subscribe Now!,” “Donate Now!,” “PayPal Donation,” and “Volunteer Now!” buttons appear above and below the picture on the left side of the screen, next to links for Facebook, Twitter, and YouTube. The links embedded in the rotating picture box include “Ask 8 Questions,” “Diseases and Vaccines,” “State Vaccine Law,” “NVIC Advocacy Portal,” “Vaccine Ingredients,” “Injury Compensation,” “Informed Consent,” “Vaccine Victim Memorial,” and “Vaccine Freedom Wall.”

Although the group states on its “About Us” webpage that it “does not advocate for or against the use of vaccines,” the preponderance of the content on its website questions the safety and efficacy of vaccines and vaccination practices, such as the CDC childhood vaccination schedule. Visitors can also download informational pamphlets designed by the organization. The downloadable literature is targeted at parents and is designed to raise questions about current vaccination practices, with emphatic titles such as “49 DOSES OF 14 VACCINES BEFORE AGE 6? 69 DOSES OF 16 VACCINES BY AGE 18? *Before you take the risk, find out what it is.*”

As an “information clearinghouse,” the NVIC website connects visitors to a diverse array of resources about vaccine safety, ranging from government agencies, such as the CDC and the Institute of Medicine, to news outlets that broadcast interviews with the group’s founder, Barbara Loe Fisher. It also provides links to vaccine advocacy events, such as Vaccine Awareness Week and antivaccination conferences.

The NVIC relies heavily on its social media outreach program, and much of its work is done through this outlet. Indeed, many of the sources on the traditional Web pages appear to be somewhat out of date, whereas its Facebook page is updated daily. In its 2011 Annual Report, NVIC states that “350,000 unique visitors accessed information on NVIC.org during FY2011,” [57], that the “Vaccine Ingredient Calculator (VIC) alone attracted more than 46,000 visits from users in 133 nations,” that its “online vaccine freedom wall saw an increase in reports of harassment by parents and health care professionals,” and that the NVIC’s “Facebook and social media outreach experience sustained growth in FY2011” [57]. Although NVIC’s traditional Web pages have as their purpose the dissemination of information about infectious disease and vaccination, the NVIC Facebook page contains posts about vaccination and other controversies in health, such as gluten allergy.

Although visitors to the NVIC website will find a great deal of governmental and scientific information on vaccines and vaccination, they are also faced with a vast number of resources that cast vaccines as dangerous. The landing page, as described previously, presents visitors with several types of information, which can make for less than straightforward navigation for the visitor seeking to learn more about a specific vaccine. The website is a repository for information on vaccine injury with links to state and federal legislation, such as the National Childhood Vaccine Injury Act of 1986, as well as links to agencies and groups that report and compensate vaccination injuries. To bolster its legitimacy, the website reflects the design choices typically employed on governmental and medical websites, replete with patriotic red, white, and blue accents on an easily legible white background, a layout resembling Vaccines.gov [55].

### Case Study 4: SANE Vax, Inc

**Figure 4:**
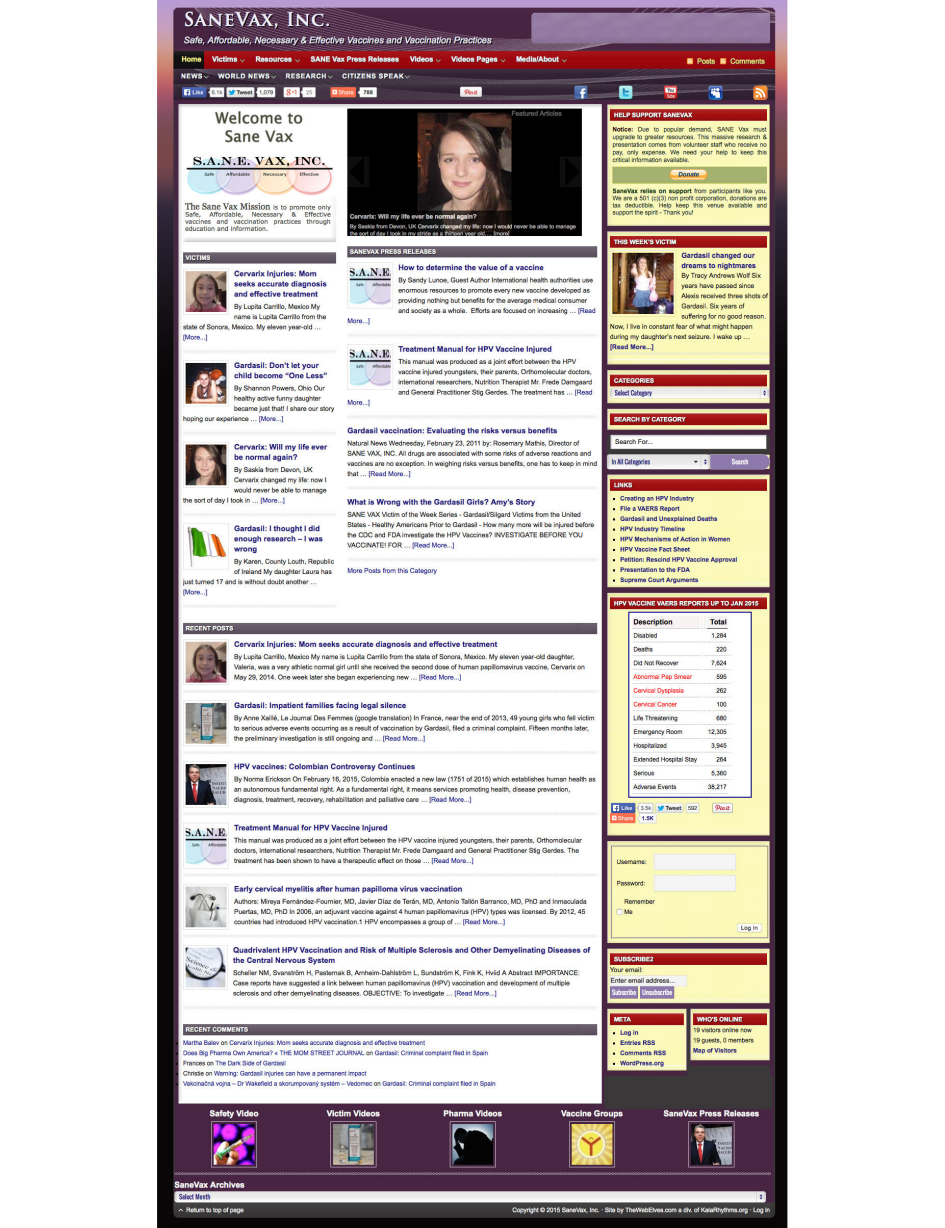
Screenshot of the SANE Vax, Inc home page.

SANE Vax states that its mission is “to promote only safe, affordable, necessary, and effective vaccines and vaccination practices through education and information” [58] (Figure 4). The nonprofit organization espouses a belief in science-based medicine and states that it offers information necessary for its visitors to make informed health decisions. Of its 5 board members, 2 state in their biographies that they are the parents of vaccine-injured children. SANE Vax presents itself as a grassroots organization in need of financial support to keep up with “popular demand” and solicits donations via a PayPal link on each page.

The majority of content on the SANE Vax website focuses on the dangers of the HPV vaccines Cervarix and Gardasil (sold in some countries as Silgard). The website’s landing page is divided into several sections corresponding to the group’s mission. Against a purple background, the upper half of the page features 2 columns: “Victims,” which is a series of short, clickable posts featuring images of vaccine-injured young women and their stories, and “SANE Vax Press Releases,” a list of position papers on vaccine policy last updated in 2011. Atop the press release column, a rotating picture box displays images of young women with narrative descriptions of their lives before and after they were vaccinated against HPV. Visitors can explore the webpage via 2 navigation bars that categorize its almost overwhelming amount of content into a series of blogs, resource pages, press release pages, and video pages. Additionally, a sidebar on the right side of the page solicits donations, displays “This Week’s Victim,” additional links to HPV-related groups, and a table listing data about HPV claims from the most recent report of the Vaccine Adverse Event Reporting System, a surveillance program sponsored by the CDC and FDA which permits anyone to submit an incident report about vaccine-related adverse effects.

There are several ways for visitors to access victims’ stories. For example, clicking on the “Victims” tab at the top of the page drops down a list of pages including a “Victims Memorial” dedicated to family remembrances of young women who died after receiving an HPV vaccine and a blog page where users submit stories about their family members who died after receiving an HPV vaccine. Most of the information on the website is text based; however, SANE Vax contains a great deal of images and videos that illustrate the stories of vaccine-injured young women. These stories tend to be narrated by family members who chronicle the young women’s healthy lives before they received the vaccination and their subsequent declines postvaccination. The text and video narratives almost always conclude by urging viewers to “investigate before you vaccinate.” The narratives are structured to juxtapose emotional appeals of vaccine injury with logical appeals to scientific research. By placing these 2 forms of persuasion side-by-side, SANE Vax achieves 2 rhetorical effects. First, it makes the argument that the scientific record is inaccurate because it omits information about vaccine injury and, second, it hopes that the viewer will place personal narratives on equal footing with scientific studies. SANE Vax’s postmodern understanding of scientific truth enables it to construct a broad digital ecology where personal truths, clinical truths, and scientific truths coexist. Although the website privileges vaccine-skeptical information, it provides a space where information can be produced and consumed in a fluid, nonhierarchical manner that, in turn, creates a more capacious understanding of vaccine and vaccine-related practices.

Like NVIC [57], SANE Vax supports a considerable digital ecology. The website links to a variety of advocacy groups, news websites, and government agencies. Keeping in-line with the website’s content, all the external information presented focuses on the dangers of vaccine and vaccination. This information comes from news reports and personal accounts from several continents giving SANE Vax a global reach, despite its status as a US nonprofit organization.

SANE Vax houses several blogs to which users can contribute after they register for a free membership to the website. The membership also permits users to upload their own text and video HPV vaccine injury narratives as well as comment others’ content. In this regard, SANE Vax creates an online community of users from around the world who share personal stories and opine about current vaccine policies.

Unlike the Vaccines.gov [55] and VEC [56] websites, SANE Vax attempts to use an exact organization scheme. According to Usability.gov, “exact organization schemes objectively divide information into mutually exclusive sections” [80]. One of the challenges this type of website organization poses to visitors of SANE Vax is that they are presented with numerous discrete categories of information on the website’s many dropdown menus. Visitors to the Vaccines.gov [55] and VEC [56] websites can access all the information about a specific disease and its vaccine with a single click, whereas visitors to the SANE Vax website are presented with information about HPV vaccines in numerous tabs and dropdown menus. The implications of this organization scheme are discussed subsequently in “User Experience.”

### Analysis

#### Ownership

Each of the websites presented in this study offered information about its sponsoring organization. Only VEC [56] and SANE Vax [58] offered biographical information about the personnel affiliated with the website and the organization it represents. Vaccines.org [55] presented disclosures of the institutions that created the content displayed on the website. As a governmental entity, it is more interested in presenting the positions of government agencies than the backgrounds of individuals holding positions within those agencies. On the other hand, the NVIC [57] “About Us” page offered no biographical data about the organization’s founder, Barbara Loe Fisher. The only mention of Fisher in this section of the website was found in a list of Frequently Asked Questions: “How do I contact NVIC, Barbara Loe Fisher, or update my contact information with NVIC?” However, Fisher’s writings and videos are showcased throughout the website.

VEC and SANE Vax disclosed their affiliated personnel’s professional achievements and personal attachments, a feature that permits users to learn more about the people presenting them with information on vaccine and vaccination-related practices. This feature also allows the website’s visitors to assess the ethos of the organizations in light of the people who founded and work for it. Although such an omission of data on Vaccines.gov is understandable because it is a convention of government agency websites, it raises questions with regard to the NVIC website. The majority of the content on NVIC was dedicated to Fisher’s advocacy work. Omitting information about her role in the organization may appear to give NVIC an official, authoritative ethos, such as that of Vaccines.gov; however, it distances the organization from the actions and positions of its founder, a rhetorical maneuver that attempts to maintain the appearance of a balanced position on vaccines and vaccination that some viewers might question.

The Vaccines.gov and VEC websites were the only 2 that included disclaimers about the medical information they presented in their “About Us” sections (and in other parts of the websites). The disclaimers functioned in 3 rhetorical ways. First, the disclaimers underscored that the information presented was not a substitute for medical treatment and opinion. These websites endorsed vaccine and vaccination. Appealing to the authority of medical practitioners demonstrated that the content presented was aligned with best medical practices. It also assumed that medical practitioners endorse vaccine and vaccination. Second, the disclaimers offered visitors a means of finding further information in the form of a medical consultation specific to their health needs. Lastly, they signified that the information presented on the website was not monolithic despite the ethos of the organizations that presented it.

#### Textual and Visual Content

All the websites in this study presented findings from the scientific literature about vaccines and vaccination. The VEC [56] and Vaccines.gov [55] websites presented the scientific information either directly or in a synthesized form and offered no further commentary on it. Thus, the logos of VEC and Vaccines.gov relied on the straightforward distribution of scientific information and governmental policies. On the other hand, NVIC [57] and SANE Vax [58] tended to present scientific information indirectly and with commentary about its quality and the conflicts of interest of its authors. It was common to find allegations on these sites that research is sponsored by the pharmaceutical industry accompanying scientific data on vaccines and vaccination.

Both NVIC and SANE Vax constructed arguments in conjunction with the presentation of scientific information and government policy. These organizations created their logos through questioning, clarifying, and challenging scientific findings. Additionally, NVIC and SANE Vax promoted alternative scientific research that accorded with their vaccine-skeptical positions. These 2 organizations constructed their “watchdog” ethos through challenging scientific and governmental knowledge and, therefore, presented counterarguments in the form of differing scientific findings and opinions on vaccine and vaccination.

Visual content played a minor role on the VEC, Vaccines.gov, and NVIC websites. Because scientific information is disseminated in text form, these websites assumed the logos, or logical argument structure, of scientific medicine even when the mission was to challenge its findings. Text-based websites are easily skimmable and searchable, aiding visitors in finding the information they seek. The encyclopedic feel of these websites added to their ethos of reputable information providers.

SANE Vax was the only website in this study that presented large amounts of visual data to its viewers. Images are the currency of Web 2.0 because they can present a large amount of information in an efficient package. SANE Vax fused its logos with the pathos of emotionally charged images that display the dangers of HPV vaccine, effectively placing scientific logic on equal ground with the personal experiences of the lay visitors and thus building a community of lay experts sympathetic to vaccine injury. Its predominately text-based counterparts in this study tended to make assertions about vaccines and vaccination based on logic and scientific reasoning. SANE Vax subverted this way of understanding vaccines by linking the faces of human suffering to vaccine. This method of argumentation requires the viewer to construct meaning in a way that differs from reading text. Reading a video image calls on the viewer’s personal knowledge, in this case about the human body and illness, thus creating a relationship between the viewer and the image. Compared to text-based reading, reading images is a more intersubjective and affective experience that makes the viewer empathize with the suffering and loss illustrated in the images. Thus, SANE Vax used pathos to build its arguments against vaccination.

As a comparison, Vaccines.gov included a few videos, but those watched by the research group seemed overly scripted and unnatural, limiting their rhetorical efficacy. This rather amateur use of video seemed half-hearted in its attempt to respond to authentic viewer concerns, presenting instead its own version of those concerns in a way that rang false. Because the videos seemed like attempts to engage viewers but were experienced as inauthentic, they not only failed to convince viewers but also diminished the ethos of the website overall.

#### Hyperlinking

The hyperlinking feature of websites (eg, its digital ecology) describes its interconnectivity with other sites. Of the 4 case studies presented, Vaccines.gov [55] contained the fewest hyperlinks to other websites. The few websites to which it linked were government agencies. The other websites in this study had considerably larger digital ecologies because they linked to numerous other websites. VEC linked to other vaccine-promoting websites, where viewers could find additional resources on special topics, such as vaccines for tween girls, and products such as provaccination children’s books. Although VEC’s [56] digital ecology may be larger than Vaccine.gov’s, its overall ecology was somewhat closed because it omitted positions on vaccines that differed from its own.

Both NVIC [57] and SANE Vax [58] linked to websites that questioned vaccine and vaccination practices as well as vaccine-related medical journal articles and government websites. Viewers navigating these websites were exposed to a variety of resources and perspectives on vaccine. NVIC and SANE Vax adopted this logical strategy to familiarize viewers with the provaccine discourses they challenged. In turn, viewers learned argumentative strategies and counterpoints to challenge the messages of websites such as VEC and Vaccines.gov. Their rhetorical strategy for hyperlinking is to demonstrate that there are many available positions on vaccines and vaccination for viewers to take. Additionally, the hyperlinking strategy demonstrated that scientific and government information is open to interpretation. In this way, NVIC and SANE Vax acknowledged the breadth and diversity of thought on vaccines and vaccination on the Internet by representing and linking to a greater diversity of positions on the subject. That is, their rhetorical ecologies were open and diverse, encouraging a variety of viewpoints even as they focused more insistently on skeptical perspectives.

#### Social Interactivity

Each of the websites offered some kind of interactive feature for viewers. Vaccines.gov [55] offered viewers surveys at the bottom of each of its pages; however, it solicited feedback to make future design and content changes to its websites. This practice is typical of US government websites. VEC [56] offered a mobile app so that viewers could reference information from its website in a smartphone-friendly format, but it did not include any social networking functions. NVIC [57] and VEC [58] displayed links to their social media accounts, where users could interact with one another. SANE Vax was the only website that permitted users to contribute their own content to its website. The website also enabled users to comment on others’ content. Contributing and commenting are 2 key community-building functions of Web 2.0 websites. The lack of interactivity of the provaccine websites seems to fit with their hierarchical understanding of scientific authority about vaccines and vaccination. The vaccine-skeptical websites allowed for more interaction and, thus, engaged the viewer in the coconstruction of knowledge about vaccination, especially with the links to social media. The most vaccine-skeptical of the websites, SANE Vax, allowed the most user engagement with content creation on the website.

As a result, SANE Vax built and supported a community of lay experts who circulated alternative knowledge about vaccines. Instead of presenting peer-reviewed scientific literature, the website created a community of peers who could view and comment on one another’s narratives. Rather than reading information on vaccines, the community members shared their experiences with vaccines, adding another level of vaccine data that Vaccines.gov and VEC could not support with their Web architecture. On the other hand, Vaccines.gov and VEC directed users to seek personal support and information from medical practitioners. These differences in interactivity clearly affect user experience and help the vaccine-skeptical websites build loyal and engaged communities, whereas the provaccine websites merely exist as online information repositories. The NVIC website occupied a somewhat middle position in this regard.

#### User Experience

All 4 of the websites in this study presented themselves to their visitors as information resources. The organizational structure of Vaccines.org [55] and VEC [56] lent itself to targeted searches about specific vaccines and vaccination-related topics. Neither of these websites offered additional commentary about vaccines and vaccination outside the realms of science and government, nor did they offer news on current events pertaining to vaccines or vaccination.

NVIC [57] and SANE Vax [58] offered many types of information about vaccines and vaccination. As a result, the websites were more difficult to navigate and their overall purposes were more difficult to discern. The NVIC website was particularly interesting with regard to purpose because it presented a more neutral position concerning vaccination on its landing page than in the rest of the website. Navigating into the site revealed deeply antivaccination sentiments that were often presented through tautological citations and links to publications by Fisher and other prominent vaccine-skeptical figures (eg, Dr Mercola of Mercola.com).

As noted previously, SANE Vax used an exact organization scheme to organize its links and information. The exact organization scheme benefits visitors looking for specific information about political action groups, manufacturers of vaccines, and victims of Gardasil in different countries. Users seeking more general information were potentially overwhelmed with scientific and lay data on vaccines, vaccine news from governments around the world, and transmissions from the website’s staff. However, SANE Vax’s design reinforced the relationship between scientific and governmental literature and personal testimony that could be used for 2 purposes. A visitor interested in personal accounts of HPV vaccine injury would find that SANE Vax’s research blogs and analyses of scientific literature reinforced the video accounts, whereas a visitor researching vaccines would find that the testimonials provided additional information to bolster SANE Vax’s claims. Thus, despite the potential confusion, SANE Vax’s architecture reflected its 2 purposes, which were to show that there are vast bodies of knowledge (in the form of personal accounts of vaccine injury) that are suppressed in the scientific and governmental literature and to demonstrate that HPV vaccines are controversial and injurious around the world. By placing personal accounts on equal footing with scientific information, the website invited visitors to share their own personal experiences in a manner that the other websites did not.

## Discussion

The provaccine websites examined in this study do not leverage the affordances of Web 2.0. The primary purpose of Vaccines.gov [55] and VEC [56] is to transmit medical and government information to viewers who are seeking specific vaccine information. Although they incorporate different types of media, those media reinforce the information-driven purposes of the websites. The unidirectional transmission of information denies viewers the opportunity to share their experiences with vaccines or to challenge the information that is presented to them. Their rhetorical ecologies are closed rather than open. The content on Vaccines.gov and VEC is vetted by physicians and government workers, but neither website acknowledges the effect that the information and policies have on the lived experiences of those who visit the websites. This unidirectional flow of information is reinforced by both websites’ hyperlinking practices. Vaccines.gov only links to other government agencies; VEC only links to provaccine websites. This practice reinforces the websites’ positions on public health while denying that there are members of the public who do not subscribe to their provaccine stances. Of course, it is not in the interest of either website to acknowledge positions that challenge their own, which may explain why neither website permits visitors to comment publicly on the information they present.

By not including interactive or community-building features on their websites, both Vaccines.gov and VEC attempt to solidify their positions as authorities on vaccines and vaccination-related practices. The obverse side of this decision is that the websites foster an image of unsympathetic authoritarians who only care about well-being at the level of the public instead of at the level of the individual. In effect, individuals whose experiences differ from the health outcomes presented on these websites have no means of interacting with those who tout vaccines and mandate vaccination practices. It is clear that many of these individuals seek an online forum where their experiences can be publicly presented and validated by a receptive community. As stated previously, according to a Pew Research’s Health Online 2013 poll, 72% of Internet users surveyed looked for health information online and 35% opted to self-diagnose with Web-based information rather than visit a clinician [16]. Considering these statistics, the lack of interactivity on the Vaccines.gov and VEC websites may turn people who have had adverse experiences with vaccines into vaccine skeptics because the only online places where their alternative experiences will be acknowledged may be vaccine-skeptical websites.

Although the quality of online vaccination information is a constant concern for researchers and practitioners, both NVIC and SANE Vax demonstrate that studies conducted in the early 2000s are inaccurate in their claims that vaccine-skeptical websites misunderstand scientific information. Rather than circulating deliberate misunderstandings of medical research, both websites strip evidence-based scientific information of its authority by questioning its primacy and call for alternative scientific studies that are sympathetic to its claims. The websites substantiate their calls for alternative research by fostering a community of individuals whose experiences with vaccines counter the information transmitted by medical and governmental websites. Through the community-building functions of Web 2.0, they curate interactive accounts of vaccine injury and skepticism, thus providing a corpus of medical texts that adhere to a different standard for scientific information; that is, the personal experience of vaccination, a purview that is absent in the information offered by Vaccines.gov and VEC.

The research presented in this study is necessarily limited because it makes case studies of only 4 of the many vaccine-related websites on the World Wide Web. However, it presents an opportunity for future research on Internet vaccine information. By employing a rhetorical framework, this study found that both provaccine websites studied concentrate on the accurate transmission of evidence-based scientific research about vaccines and government-endorsed vaccination-related practices. On the other hand, the vaccine-skeptical websites investigated focus on creating communities of people affected by vaccines and vaccine-related practices. From this more personal framework (see also Lawrence et al [49]), the websites then challenge the information presented in scientific literature and government documents. At the same time, the vaccine-skeptical websites in this study are repositories of vaccine information and vaccination-related resources.

Future studies on vaccination and the Internet should take into consideration the rhetorical features of provaccine and vaccine-skeptical websites and further investigate the role of Web 2.0 community-building features on vaccine-related practices. More work needs to be done to determine if the findings of this small pilot study can be replicated across more provaccine and vaccine-skeptical websites; that is, whether the features identified here are generalizable.

## Abbreviations

CDC: Centers for Disease Control
CHOP: Children’s Hospital of Philadelphia
FDA: Food and Drug Administration
HHS: Health and Human Services
HPV: human papillomavirus
MMR: measles, mumps, rubella
NVIC: National Vaccine Information Center
NVPO: National Vaccine Program Office
VEC: Vaccine Education Center
VIC: Vaccine Ingredient Calculator
